# Chitin synthesis and fungal pathogenesis

**DOI:** 10.1016/j.mib.2010.05.002

**Published:** 2010-08

**Authors:** Megan D Lenardon, Carol A Munro, Neil AR Gow

**Affiliations:** Aberdeen Fungal Group, School of Medical Sciences, Institute of Medical Sciences, University of Aberdeen, Aberdeen, AB25 2ZD, UK

## Abstract

Chitin is an essential part of the carbohydrate skeleton of the fungal cell wall and is a molecule that is not represented in humans and other vertebrates. Complex regulatory mechanisms enable chitin to be positioned at specific sites throughout the cell cycle to maintain the overall strength of the wall and enable rapid, life-saving modifications to be made under cell wall stress conditions. Chitin has also recently emerged as a significant player in the activation and attenuation of immune responses to fungi and other chitin-containing parasites. This review summarises latest advances in the analysis of chitin synthesis regulation in the context of fungal pathogenesis.

## Introduction

Chitin is an essential component of the cell walls and septa of all pathogenic fungi, and occurs in the cyst walls of pathogenic amoebae, the egg-shells and gut lining of parasitic nematodes and the exoskeletons of invertebrate vectors of human disease including mosquitoes, sand flies, ticks and snails. Despite the fact that chitin is only outweighed in abundance in nature by cellulose and is present and essential in so many parasites and pathogens, fundamental information about its biosynthesis and recognition by the immune system is lacking.

**Figure 1:**
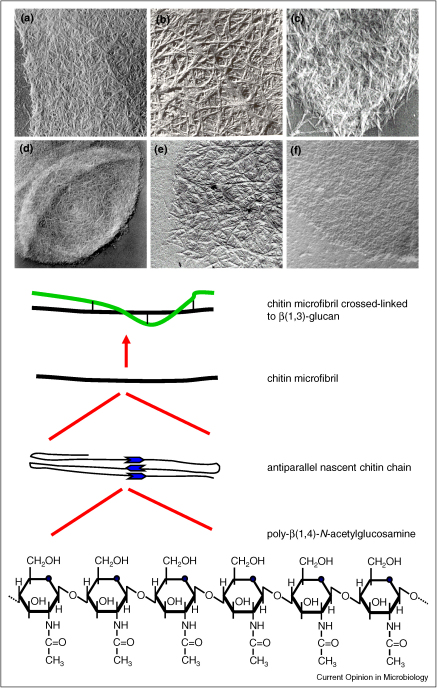
Chitin structure and diversity in fungi. Chitin is a β(1,4)-homopolymer of *N*-acetylglucosamine that folds in an anti-parallel manner forming intra-chain hydrogen bonds. Chitin chains are cross-linked covalently to β(1,3)-glucan (green) to form the inner skeleton of most fungi. Examples of shadow cast electron microscopy images of chitin from **(a)***Neurospora crassa*; **(b)***Coprinus cinereus*; **(c)** chitin–chitosan from *Mucor mucedo*; and **(d)***Candida albicans*. In **(e)** and **(f)**, the structure of chitin from *C. albicans* is shown in a *chs3*Δ and *chs8*Δ mutant, respectively, demonstrating that the architecture of chitin is genetically determined [24].

Chitin, a β(1,4)-linked homopolymer of *N*-acetylglucosamine, is a simple polysaccharide that is represented in the cell walls of all fungi studied to date [1,2]. The nascent primary polysaccharide of fungi folds back on itself to form anti-parallel chains, which form intra-chain hydrogen bonds that further stiffen the carbohydrate into immensely strong fibrous microfibrils tougher than any other molecule in nature, and stronger, weight-for-weight, than bone or steel (Figure 1). The 3D network of chitin microfibrils is covalently attached to β(1,3)-glucan — a second load-bearing polysaccharide present in most fungal cell walls [3]. In many species, such as the human pathogen *Candida albicans*, the major classes of cell wall proteins are attached through a GPI-remnant to β(1,3)-glucan or chitin via a branched β(1,6)-glucan linker [3]. A variable proportion of fungal chitin is synthesised and then deacetylated to chitosan by the action of one or more chitin deacetylases. In *C. albicans*, less than 5% of chitin is deacetylated to chitosan, while most zygomycetes and the basidomycete *Cryptococcus neoformans* have more than two thirds deacetylated to chitosan [4,5]. Chitin deacetylation may make the polymer more elastic and protect it from the action of hostile chitinases.

The apparent simplicity of the chitin primary structure belies a complex underlying biosynthetic process. Chitin is synthesised by large families of chitin synthase (CHS) enzymes that fall into seven discernable classes (Table 1). We have chosen to follow the classification proposed by Niño-Vega *et al.* [6] and Roncero [7], and not that of Chigira *et al.* [8] and Choquer *et al.* [9] who reverse classes VI and VII. The functional significance of all CHS classes is not clear and seems to differ in different fungi [1,2]. The roles of individual CHS genes have been investigated principally by analysis of specific CHS deletion strains. Class I enzymes are most readily measured in *in vitro* biochemical assays, yet they normally make only a minor fraction of cell wall chitin, and mutants lacking class I CHS genes are invariably viable with mild phenotypes under non-stressed conditions [1]. Class II enzymes often are immeasurable in enzyme assays, and make little chitin, but their deletion results in marked deleterious effects on cell viability through effects on vital processes such as primary septum formation [10]. Class IV enzymes often make substantial amounts of wall chitin, but mutants are usually viable although sometimes attenuated in virulence [11]. Class IV, V and VII sequences share some sequence homology, and class V and some class VII proteins also contain myosin-like domains. Class III, V, VI and VII have only been identified in filamentous fungi and some dimorphic fungi and are absent from yeasts like *Saccharomyces cerevisiae* and *C. albicans*.

The multiplicity of CHS enzymes suggests that they may have redundant roles in cell wall synthesis. This is the case for some but not all CHS enzymes. It is clear that the expression and activity of CHS is highly regulated both throughout the cell cycle and under conditions of stress, such as in response to potentially lethal challenges to cell integrity imposed by lytic enzymes or antibiotics or oxidants that are generated by the respiratory burst within the phagolysomes of lymphocytes.

## Transcriptional regulation of chitin synthesis

In most fungi, chitin and cell wall synthesis occurs at sites of polarised growth. During early bud growth, cell wall material is deposited at the bud tip [12]. A period of isotropic growth occurs in large budded cells where material is deposited over the entire bud surface. Following nuclear division, a repolarisation phase begins where material is directed towards the mother-bud neck to prepare for cytokinesis. In hyphal or filamentous forms, cell extension is a continuous and indefinite process of apical growth [13].

Accordingly, chitin synthesis must be regulated both temporally and spatially in relation to the cell cycle. The *S. cerevisiae* class II enzyme *Sc*Chs2 synthesises the primary septum and *Sc*Chs1 of class I acts a repair enzyme that replenishes chitin in the birth/bud scar immediately after cytokinesis [1,14]. A genome-wide analysis of cell cycle regulation at the mRNA level using synchronised *S. cerevisiae* cultures indicated that expression of *ScCHS2* peaked in M-phase and *ScCHS1* in M/G1, both at appropriate times for the functions of these enzymes [15]. Transcription during the cell cycle of an opaque *C. albicans MTL*a *FAR1*^OP^ strain synchronised after release from α-factor arrest showed that expression of *CaCHS1* (orthologous to *ScCHS2*), *CaCHS8* and to a lesser extent *CaCHS3* peaked in G2 phase, whereas expression of *CaCHS2* was non-periodic [16^•^].

**Figure 2:**
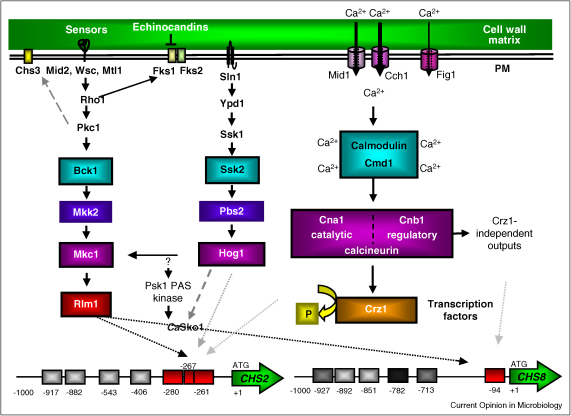
Signalling pathways that regulate *Candida albicansCHS* gene expression. The HOG, PKC and the Ca^2+^/calcineurin signalling pathways regulate chitin synthesis and *CHS* gene expression [20]. The Rlm1 transcription factor downstream of the PKC MAP kinase cascade controls the expression of a number of cell wall related genes in *S. cerevisiae*. Putative Rlm1 binding motifs (red boxes) in the promoters of *CaCHS2* and *CaCHS8* contribute to their cell wall stress-activated regulation [21]. Activation of the calcineurin pathway results in de-phosphorylation of the *Ca*Crz1 transcription factor. *Ca*Crz1 then moves to the nucleus and induces expression of genes with CDREs (calcium-dependent response elements) within their promoter sequences. *C. albicans* has significant re-wiring of signalling pathways compared to *S. cerevisiae*, for example, the role of the *Ca*Sko1 transcription factor in response to caspofungin is independent of the *Ca*Hog1 MAP kinase but involves the *Ca*Psk1 PAS kinase [26]. Potential CDREs and Sko1 binding sites identified *in silico* upstream of *CaCHS2* and *CaCHS8* (grey boxes) were not required for regulation of gene expression [21]. Sequences in the first 347 bp and 125 bp of the *CaCHS2* and *CaCHS8* promoters governed expression through these three signalling pathways [21].

Adapted from [17].

Disruption of cell wall biosynthetic genes or treatments with cell wall perturbing agents often results in compensatory alterations in the cell wall, including activation of chitin synthesis, in an attempt to maintain cellular integrity (reviewed in [17]). Defects in the cell wall are sensed in *S. cerevisiae* by transmembrane proteins such as *Sc*Wsc1 and *Sc*Mid2, or the signalling mucins *Sc*Msb2 and *Sc*Hkr1 that activate downstream mitogen-activated protein (MAP) kinase cascades to bring about cell wall remodelling (Figure 2). In *S. cerevisiae* this so-called ‘cell wall salvage’ or ‘cell wall compensatory’ pathway is mediated primarily through the protein kinase C (PKC) cell integrity MAP kinase cascade and its downstream target the transcription factor *Sc*Rlm1 [18]. A second MAP kinase cascade, the high osmolarity glycerol response (HOG) pathway, has also been suggested to play a role in regulating cell wall architecture [19] (Figure 2).

In *C. albicans*, the PKC and HOG MAP kinase cascades and the Ca^2+^/calcineurin pathway regulate CHS gene expression and chitin synthesis in response to cell wall stresses [20]. Promoter dissection experiments have defined the regulatory regions of the class I CHS promoter sequences and revealed that *C. albicans* uses different transcription factors and/or consensus binding sequences to regulate chitin synthesis compared to *S. cerevisiae* [21] (Figure 2).

Upregulation of chitin synthesis in response to cell wall stress may be clinically relevant. In *C. albicans*, the transcriptional activation of chitin synthesis through the PKC, HOG and Ca^2+^/calcineurin pathways confers resistance to the echinocandin class of antifungal drugs [22^•^], and in *Aspergillus fumigatus*, calcineurin-mediated elevated transcription of *AfCHSA* and *AfCHSC* is required for paradoxical growth in the presence of high concentrations of the echinocandin drug caspofungin [23^••^].

## Post-transcriptional regulation of chitin synthesis

Coordinated synthesis of chitin also requires the localisation of the enzymes to be regulated throughout the cell cycle. Fungal CHS enzymes that are responsible for synthesising cell wall and/or septal chitin tend to be localised to sites of polarised growth. For example, in *C. albicans*, the class IV enzyme *Ca*Chs3 is involved in both cell wall and septum synthesis [11]. It is localised to the tip of growing buds and hyphae and relocates to sites of septum formation before cytokinesis [24]. Similarly, in *A. nidulans*, the class III enzyme *An*ChsB appears to function at polarised growth sites and in forming septa during hyphal growth and conidia development [25].

**Figure 3:**
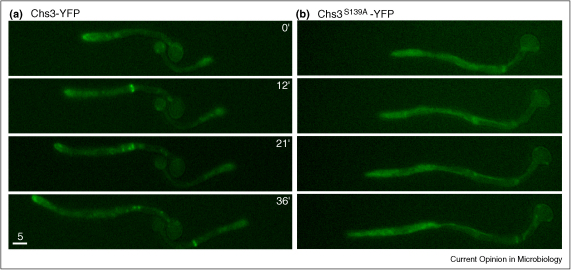
Phosphorylation of *Candida albicans* Chs3 on a specific serine residue is required to target the CHS to sites of polarised growth. *Ca*Chs3 was tagged with yellow-fluorescent protein (YFP) and the localisation of the CHS in growing hyphae was observed by time-lapse fluorescence microscopy. **(a)***Ca*Chs3-YFP localises to the tip of the growing hypha and then flashes at the site of septum formation. **(b)** Mislocalisation of *Ca*Chs3 is observed when the serine at position 139 has been mutated to an alanine that cannot be phosphorylated (Chs3^S139A^-YFP).

Modified from [30^•^].

Both the localisation and stability of CHS enzymes can be regulated by phosphorylation. Recent work has shown that protein kinases and the establishment of cell polarity appear to be important for cell wall regulation in *C. albicans* [26]. In *S. cerevisiae*, the primary septum is synthesised by *Sc*Chs2 [1,14]. Phosphorylation of *Sc*Chs2 by *Sc*Cdk1 at 4 N-terminal sites retains this enzyme in the Endoplasmic Reticulum (ER) until mitotic exit [27]. Deletion of these phospho-sites resulted in *Sc*Chs2 degradation [28], indicating that phosphorylation regulates chitin synthesis by *Sc*Chs2 at specific stages of the cell cycle, either by regulating the cellular localisation or stability of the protein. Three of the four CHS enzymes are phosphorylated in *C. albicans* [29], and phosphorylation of *Ca*Chs3 on S139 is required to target this enzyme to sites of polarised growth [30^•^] (Figure 3).

The regulation of localisation of the class IV CHS enzyme in *S. cerevisiae* has been studied extensively and involves a number of different post-transcriptional regulatory mechanisms. Export of *Sc*Chs3 from the ER is controlled by the chaperone *Sc*Chs7 and transportation from the Golgi to the plasma membrane (PM) occurs in specialised vesicles called chitosomes [31,32]. Generation of the PtdIns(4)P lipid by *Sc*Pik1 promotes forward transport of *Sc*Chs3 to the PM and subsequent de-phosphorylation by *Sc*Sac1 terminates the signal, allowing *Sc*Chs3 to remain in the PM and synthesise chitin [33]. *Sc*Chs3 is targeted to the bud neck for septum formation at an appropriate time in the cell cycle through interactions with *Sc*Chs4, *Sc*Bni4, *Sc*Glc7 and the septins [14,34–38].

In addition to mechanisms involving post-translational modifications or protein–protein interactions, some CHSs are hybrid proteins with N-terminal myosin motor-like domains (MMD) and C-terminal CHS domains. These enzymes tend to fall into classes V and VII, and the best characterised examples from human pathogens include *Wd*Chs5 (the class V enzyme from *Wangiella dermatitidis*; [39]), *An*CsmA and *An*CsmB (class V and VII from *A. nidulans*; [40,41]). Using their MMD, these enzymes appear to localise themselves to sites of polarised cell wall expansion in an actin-dependent manner [40,42–44]. The MMD may not possess the motor activity of traditional myosins [43], however, in the plant pathogen *Ustilago maydis*, the myosin-like domain of the class V *Um*Mcs1 enzyme has been shown to be essential for its apical localisation and is involved in retention of *Um*Mcs1 in the apical dome (G Steinberg, personal communication).

## Chitin and immune recognition

The immune system has evolved to detect conserved, basic molecular components of microorganisms called pathogen associated molecular patterns (PAMPs). β(1,3)-Glucan is a well studied PAMP that is detected by Dectin-1, a C-type lectin receptor of monocytes and macrophages, and is of major importance in activating a strong, pro-inflammatory response by the innate immune system [45]. In *C. albicans*, access to the inner cell wall layer containing β-glucan and chitin is normally shielded by the superficial mannans and so immune detection of intact cells is initially focused on mannan-immune receptor interactions [46]. However, β-glucan can be unmasked by exposure to host enzymes, antifungal drugs, heat-treatment and in mannosylation deficient mutants [47]. In a similar way, an outer α(1,3)-glucan layer masks detection of the inner chitin/β-glucan layer in *Histoplasma capsulatum* [48]. In the case of β-glucan, unmasking has major immunomodulatory consequences. What therefore is the immunological role of chitin, the other highly conserved signature molecule in the inner cell wall?

Recent studies have begun to reveal a complex picture regarding the immunological properties of chitin [49]. Immune responses seem to be highly dependent on the size of the chitin fragments used to stimulate immune cells [50]. Very large (>100 μm) chitin fragments, normally prepared from invertebrate sources, seem to be immunologically inert, while intermediate (40–70 μm) and small chitin (<40 μm) seem capable of activating macrophages and eliciting IL-17, TNF and IL-23 production via a range of pattern recognition receptors (PRRs) [51]. All of these particles are quite large relative to the sizes of fungal cells and the molecular scale of immune receptor–ligand interactions. Also, such experiments have yet to accommodate the fact that the structure of chitin microfibrils varies significantly in different organisms and even in different parts of the cell wall [24] (Figure 1). Size-dependent immune reactivity helps explain the importance of chitin-degrading proteins, such as acidic mammalian chitinase [52] and chitotriosidase [53,54] in allergic immune responses. These enzymes presumably digest chitin into smaller particles, capable of mediating allergic responses. Their ability to digest large chitin fragments results in the accumulation of IL-4 expressing basophils, eosinophils and neutrophils in tissues and induces alternative macrophage activation that is associated with the immunity to certain chitin-containing parasites such as the helminth *Nippostrongylus brasiliensis* [55^••^]. Reciprocally, it appears as though certain human proteins sequester chitin to dampen immune responses [56].

Recently, several candidate mediators of chitin mediated immune responses have been identified. RegIIIg (HIP/PAP) is a C-type lectin with an affinity for chitin that is expressed in the neutrophil-like Paneth cells of the small intestine and is induced by certain bacterial peptidoglycans [57]. Peptidoglycans are chemically related to chitin in so far as they are *N*-acetylglucosamine containing polysaccharides and the two molecules can activate some common cellular responses [58]. FIBCD1 has also been identified as a calcium-dependent acetyl group-binding tetrameric chitin-binding receptor [59^••^]. The binding between this chitin receptor and acetylated BSA could be inhibited by a variety of acetylated compounds, but not glucosamine or glucose [59^••^]. The requirement for acetylation suggests that this receptor is unresponsive to chitosan, which is known to be able to activate dendritic cells via a TLR4-dependent mechanism [60]. This is important because fungal pathogens such as the zygomycetous fungi and *C. neoformans* have substantial amounts of deacetylated chitin in their walls. Therefore further chitin and chitosan immune receptors remain to be identified and the role of fungal chitin in immune recognition requires investigation.

## Chitin as a target for anti-fungal chemotherapy

Chitin and chitosan are hallmark polysaccharides that are present in all known fungal pathogens and not in humans. Inhibition of chitin synthesis has therefore been proposed as an attractive target for antifungal therapies. However, no CHS inhibitor has ever progressed into clinical practice [61]. Existing CHS inhibitors such as the nikkomycins and polyoxins are most potent and specific against class I enzymes but are less effective inhibitors of other classes of CHS enzymes, and of fungal growth *in vivo* [61,62].

The discovery of the essential role of *C. albicans CHS1* [10] prompted the screening for class II CHS inhibitors. Roche developed one compound, RO-09-3143, a specific inhibitor of *Ca*Chs1 that was fungistatic to *C. albicans*, but cidal in a *chs2*Δ mutant background [63]. This highlighted the possibility of compensatory functions for different CHS enzymes and was reinforced by the observation that *C. albicans* yeast cells stimulated to synthesise chitin by treatment with Ca^2+^ and Calcofluor White were able to grow and divide by forming a novel salvage septum in the absence the otherwise essential *CaCHS1* gene [22^•^].

A common response to cell wall damage is strengthening of the wall by the production of excess chitin, primarily by the class IV enzymes such as *Sc*Chs3 and *Ca*Chs3 [20,64]. This compensatory mechanism is triggered when fungi are treated with echinocandin drugs [22^•^,65,66]. Enhanced chitin levels reduces susceptibility to echinocandin drugs in *C. albicans* [17,22^•^], but reciprocally, combinations of CHS and glucan synthase inhibitors are more potent against *C. albicans* and *A. fumigatus* than individual drug treatments [22^•^,67]. These studies highlight the potential of combination therapies that target the synthesis of the two major structural polysaccharides found in most fungi, in achieving fungicidal regimens that would prevent the emergence of resistance mechanisms. In addition, cell wall synthase inhibitors, applied in combination with antagonists of the signalling pathways that regulate synthase expression and activity, may have potential as potent antifungal combination therapies. For example, the calcineurin pathway is important for regulation of chitin synthesis in *C. albicans* and *A. fumigatus* as well as the response to echinocandin drugs, and drugs that block the calcineurin pathway act synergistically with the echinocandins [20,22^•^,67,68].

## Conclusions

The primary structure of chitin comprises a single sugar type and a single inter-sugar linkage. None-the-less it is diverse in structure and form and is assembled by different classes of enzymes encoded by families of genes whose expression is regulated in a cell cycle-dependent manner at the transcriptional and post-transcriptional levels. Recent advances in our understanding of how chitin synthesis is regulated and responds to cell wall stress sustains the attraction of this process as a potential antifungal drug target, possibly in combination with inhibitors of β(1,3)-glucan synthesis and/or the cell wall compensatory pathway. The relevance of fungal chitin synthesis in human disease is also evident in emerging research defining the role of this fungal signature molecule in immune recognition mechanisms.

## References and recommended reading

Papers of particular interest, published within the annual period of review, have been highlighted as:

• of special interest
•• of outstanding interest

## Figures and Tables

**Table 1:** Members of the seven classes of CHS enzymes in various fungi^a^

Fungus^b^	I	II	III	IV	V	VI	VII	Total
*S. cerevisiae*^d^	Chs1	Chs2		Chs3				3
*C. albicans*^d^	Chs2 Chs8	Chs1		Chs3				4
*A. nidulans*^d^	ChsC	ChsA	ChsB ChsF	ChsD	CsmA	ChsG	CsmB	8
*A. fumigatus*^d^	ChsA	ChsB	ChsC ChsG	ChsF	ChsE	ChsD	Afu2g13430	8
*W. dermatitidis*^d^	Chs2	Chs1	Chs3	Chs4	Chs5	Chs6	Chs7	7
*U. maydis*^e^	Chs3 Chs4	Chs2	Chs1	Chs5 Chs7	Mcs1		Chs6	8
*C. neoformans*^e^	Chs6 Chs8	Chs6 Chs8	Chs2 Chs7	Chs1 Chs3	Chs5		Chs4	8
*P. blakesleeanus*^f^	Chs5 Chs6	Chs1 Chs2 Chs3 Chs4		Chs7 Chs8 Chs9 Chs10				10^c^

a Standard genetic nomenclature for *S. cerevisiae* and *C. albicans* has been used to designate CHS proteins from all fungi. The enzymes have been assigned to classes based on the classification proposed by Niño-Vega *et al.* [6] and Roncero [7].

b d: ascomycete; e: basidomycete; and f: zygomycete.

c 34 putative CHSs have been annotated in the *P. blakesleeanus* genome (http://genome.jgi-psf.org/Phybl2/Phybl2.home.html).
